# Comparison of positive BioFire FilmArray meningitis/encephalitis (ME) panels, CSF cultures, CSF parameters, clinical presentation and in-patient mortality among patients with bacterial and fungal meningitis

**DOI:** 10.1128/spectrum.00014-24

**Published:** 2024-12-23

**Authors:** Thein Myint, Jaime Soria, Yuanzheng Gao, Marice Ruiz Conejo Castillo, Vaneet Arora, Julie A. Ribes

**Affiliations:** 1 Division of Infectious Diseases, University of Kentucky, Lexington, Kentucky, USA; 2 Department of Pathology, Cooper University Health Care Allied Health Programs, Camden, New Jersey, USA; 3 CrescentCare, New Orleans, Louisiana, USA; 4 Department of Pathology and Laboratory Medicine, University of Kentucky, Lexington, Kentucky, USA; Ann & Robert H. Lurie Children's Hospital of Chicago, Chicago, Illinois, USA

**Keywords:** meningitis/encephalitis (ME) panels, BioFire, positive predictive value

## Abstract

The BioFire FilmArray meningitis/encephalitis panel (MEP) was brought to the University of Kentucky in 2016 to aid in the identification of community-acquired meningitis and encephalitis (ME). This panel has shown variable performance with some institutions showing high sensitivity and specificity for many pathogens but others seeing false positives during clinical use. We evaluated the panel’s performance using retrospective chart review of patients at the University of Kentucky from October 2016 to September 2022, including 7,551 MEP results. Cerebrospinal fluid (CSF) samples with positive results for bacterial and fungal pathogens were compared with CSF and blood cultures, other laboratory parameters, and clinical presentations, to classify MEP results as true positive (TP), likely TP, or false positive (FP). Of 132 patients with positive bacterial or fungal MEP results, 48.9% of bacterial and 88.9% of cryptococcal analytes were classified as TP. The positive predictive value (PPV) varied by organism, with the highest being *Listeria monocytogenes* and *Neisseria meningitidis*. One-third of the FP results were *Streptococcus agalactiae*. Among patients with likely TP, 75.7% (28 out of 37) received oral or IV antibiotics before blood or CSF culture. MEP was 100% specific compared to culture. The PPV based on pathogen ranged from 78.6% to 100%. Interestingly, 27.4% (29 out of 106) cases (excluding FPs) would have no pathogen definitively identified if MEP had not been used. This study highlights the utility of MEP in rapidly diagnosing ME, particularly in patients pretreated with antibiotics. It also emphasizes the importance of correlating MEP results with clinical assessments and other diagnostic tests to assure accuracy.

**IMPORTANCE:**

This study compares the performance of the meningitis/encephalitis panel (MEP) in detecting bacterial and fungal pathogens with cerebrospinal fluid cultures and other parameters. Almost half of bacterial analytes of MEP had positive cerebrospinal fluid (CSF) or blood cultures; the remaining 42% of bacterial analytes were correlated with clinical presentation and other CSF parameters. 27.4% (29 out of 106) cases would not have had a pathogen definitively identified if the MEP had not been used. This study highlights the importance of using MEP as a diagnostic tool, especially in patients who have already received antibiotics, where traditional culture-based methods may not be diagnostic. This research underscores the use of MEP in improving the speed of diagnosing meningitis. However, it emphasizes that MEP can produce false positive results in some patients. It is therefore necessary to interpret MEP results together with clinical assessments and other diagnostic tests to ensure the most accurate diagnosis.

## INTRODUCTION

Diagnosing bacterial and fungal meningitis is challenging due to the urgency of treatment, overlapping symptoms with other conditions, and limitations of current diagnostic methods. The diagnosis of bacterial meningitis typically relies on blood and cerebrospinal fluid (CSF) cultures, Gram staining, and bacterial antigen detection (1, 2).

Elevated CSF white blood cell count, low glucose, and high protein levels can also be used to suggest bacterial meningitis, but these are poor indicators of viral encephalitis or meningitis. Traditional culture methods, although the gold standard, take days to yield results. Prior antibiotic use, while lifesaving, can reduce CSF culture yields and complicate CSF interpretation (3, 4).

Newer diagnostic techniques, such as polymerase chain reaction (PCR) assays and multiplex PCR panels, provide faster results and detect a broader range of pathogens. The BioFire FilmArray meningitis/encephalitis panel (MEP) was the first multiplex PCR approved by the FDA in October 2015 to evaluate CSF samples for community-acquired meningitis syndromes. The MEP can identify 14 organisms in a single test reaction, including six bacteria, one yeast, and seven viral agents of meningitis (5).

Several studies have highlighted the clinical impact of the MEP on patient care. The panel has influenced antimicrobial therapy decisions and duration, leading to more targeted and effective treatment strategies (6, 7). The rapid run time of approximately 1 h for results is a significant advantage, allowing prompt and targeted treatment decisions to be made (8). The use of MEP has also been shown to reduce the average length of stay in one study (3).

While the MEP has demonstrated high diagnostic accuracy for many pathogens, there have been reports of limitations, such as false-negative results for specific targets such as *Cryptococcus neoformans*. (9, 10) Additionally, some studies have reported unexpectedly high false-positive rates for some of the bacterial pathogens, such as *Streptococcus pneumoniae*, *Streptococcus agalactiae* (11), and *Haemophilus influenzae* (12), which warrants careful correlation of these positive results with other diagnostic features. Further research and ongoing evaluation of the panel’s performance in diverse clinical settings are needed to refine its role in diagnosing and managing central nervous system infections. By delving into the performance of the MEP in a tertiary care center in central Kentucky, this research aims to add to the growing body of evidence of the panel’s role in enhancing diagnostic capabilities and optimizing patient outcomes in the context of central nervous system infections.

## MATERIALS AND METHODS

### Study design and data collection

A retrospective chart review was conducted for patients admitted for the evaluation of central nervous system infection at the University of Kentucky Healthcare Center from October 2016 to September 2022. We collected demographic data, clinical presentation, and test indication if bacteria or fungus was identified. Results of the MEP, CSF, blood cultures, and CSF cellularity, protein, and glucose were collected using an electronic medical record search.

### Pathogen selection and analysis

The performance of the MEP for detecting six bacterial pathogens and one fungal pathogen was analyzed. Viral pathogens in the MEP were not included in this study since separate CSF viral PCRs or CSF viral cultures were not routinely ordered at the University of Kentucky, and the viral culture yield was unreliable as a mechanism of confirmatory testing. The MEP bacterial or fungal result was considered a true positive (TP) if it was confirmed by CSF culture. It was classified as a likely TP based on a positive CSF Gram stain, blood culture, and other CSF findings consistent with clinical meningitis. It was considered a false positive (FP) if the MEP was discordant with CSF and clinical findings. The sensitivity, specificity, and positive and negative predicted values (PPV and NPV) were calculated based on these correlations. The clinical characteristics, outcomes, and CSF findings of different pathogens among patients with positive MEP were also compared.

### Statistical analysis

Descriptive statistics were employed to analyze the clinical and epidemiological features. Fisher’s exact test was used to compare categorical variables. The Kruskal-Wallis test was used to compare continuous variables without a normal distribution. The analysis involves pairwise comparisons among the columns, not against a single gold standard. For multiple comparisons, Fisher’s exact test with Bonferroni adjustment was used for categorical variables, while the Dunn test was performed for continuous variables. Shapiro-Wilk test was used to assess the normal distribution. Statistical analyses were performed with Stata 17 (College Station, TX: Stata Corp LLC).

## RESULTS

A total of 7,551 panels from 6,943 patients were included in the analysis. The median age for the entire cohort was 18.5 years old (interquartile range [IQR]: 0–34 years old), and 53% were male. The majority of patients were White (85.9%), 7.9% were Black, 2.9% were Hispanic, 0.7% were Asian, and 2.6% were unreported. About 132 (1.7%) panels were positive for bacterial or fungal pathogens, with 17 remaining positive on consecutive testing. These repeat results were excluded from statistical analysis. Clinical characteristics, outcomes, and CSF findings for 115 patients with positive MEP for bacterial and fungal organisms are shown in Table 1. There were 88 patients with bacterial meningitis and 27 patients with cryptococcal meningitis. *S. pneumoniae* was the most common bacterial pathogen identified by the MEP (*N* = 41 patients). The average turnaround time for the results was 121 min from the arrival in the laboratory (range: 60–316 min).

**TABLE 1:** Clinical characteristics, outcomes, and CSF findings of patients with positive BioFire FilmArray ME panels^*a*^

	*Streptococcus pneumoniae* *n* = 41	*Streptococcus agalactiae* *n* = 14	*Haemophilus influenzae* *n* = 16	*Escherichia* *coli* *n* = 11	*Listeria* *monocytogenes* *n* = 5	*Neisseria meningitidis* *n* = 1	*Cryptococcus neoformans* *n* = 27	*P* value^*b*^
Age	47y [11–55y]	9w [5w–16y]	5y [1–46y]	5w [3–6w]	32y [5–55y]	18y	49y [35–63y]	<0.001
Sex, male	27 (65.9)	9 (64.3)	8 (50.0)	9 (81.8)	1 (20.0)	0 (0.0)	21 (77.8)	1.000
Race, white	35 (85.4)	11 (78.6)	16 (100.0)	9 (81.8)	6 (100.0)	1 (100.0)	26 (96.3)	1.000
Race, black	5 (12.2)	2 (14.3)	0 (0.0)	2 (18.2)	0 (0.0)	0 (0.0)	1 (3.7)	1.000
Positive blood culture	17/40 (42.5)	6/12 (50.0)	5/15 (33.3)	3/9 (33.3)	0/4 (0.0)	0/1 (0.0)	6/26 (23.1)	1.000
Positive CSF culture	22 (53.7)	5 (35.7)	6 (37.5)	6 (54.5)	4 (80.0)	0 (0.0)	24 (88.9)	0.021
Positive Gram stain	27 (65.9)	5 (35.7)	8 (50.0)	4 (36.4)	3 (50.0)	0 (0.0)	13 (48.1)	1.000
Positive India ink							13 (48.1%)	NA
Positive blood, CSF culture or Gram stain	33 (80.5)	9 (64.3)	8 (50.0)	7 (63.6)	4 (66.7)	0 (0.0)	25 (92.6)	0.252
CSF cell count (cells/μL)	1,848 [314–6,733]	34 [11–211]	841 [60–1,780]	259 [91–583]	514 [149–925]	135	44 [15–124]	<0.001
CSF protein (mg/dL)	328 [145–548]	152 [46–335]	119 [66–250]	281 (230–335)	203 [149–250]	62	110 [78–140]	<0.001
CSF glucose (mg/dL)	21 [2–53]	57 [18–61]	38 [8–64]	35 [3–45]	37 [27–66]	63	28 [7–54]	0.171
Immunocompromised	5 (12.2)	0 (0.0)	0 (0.0)	0 (0.0)	0 (0.0)	0 (0.0)	14 (51.9)	0.021
Mortality	8 (19.5)	2 (14.3)	2 (15.3)	0	1 (20.0)	0 (0.0)	9 (33.3%)	1.000

^*a*^ Continuous variables are expressed as median [IQR]. Categorical variables are expressed as frequency (percentage).

^*b*^ Fisher’s exact test with Bonferroni adjustment for categorical variables. Dunn test for continuous variables.

The median age of diagnosis varied significantly between different pathogens, with those transmitted post-partum presenting within several weeks of birth (5 weeks for patients with *Escherichia coli* and 9 weeks for patients with *S. agalactiae*), while patients with *S. pneumoniae* and *C. neoformans* were diagnosed at a median age of 47 and 49 years, respectively (*P* < 0.001).

The detection rate of blood culture with the same etiological agent as in MEP varied. The highest rate was among patients with *C. neoformans* at 88.9% (24 out of 27). The blood culture detection rate was lower among patients with bacterial meningitis: 50% (6 out of 12) for *S. agalactiae*, 42.5% (17 out of 40) for *S. pneumoniae*, 33.3% (5 out of 15) for *H. influenzae,* and 33.3% (3 out of 9) for *E. coli*. Negative blood cultures were seen in all patients in this study who were infected with *L. monocytogenes* or *N. meningitidis*.

The CSF culture positivity rate was also the highest among patients with *C. neoformans* at 88.9% (24 out of 27). It was slightly lower for bacterial meningitis: 80% (4 out of 5) for *L. monocytogenes*, 54.5% (6 out of 9) for *E. coli*, 53.7% (22 out of 40) for *S. pneumoniae*, 37.5% (6 out of 15) for *H. influenzae*, 35.7% (5 out of 14) for *S. agalactiae,* and 0% (0 out of 1) for *N. meningitidis*.

Regarding CSF parameters, the median CSF cell count and protein were higher in patients with *S. pneumoniae* meningitis and lower for CSF glucose than the rest of bacterial and fungal MEP. The median CSF cell count was 1,848 /µL for *S. pneumoniae* meningitis, 841 /µL for *H. influenzae*, 514 /µL for *L. monocytogenes*, 259 /µL for *E coli*, 135 /µL for *N. meningitidis*, 44 /µL for *C. neoformans*, and 34 /µL for *S. agalactiae* meningitis. The median CSF protein was 328 mg/dL for *S. pneumoniae*, 281 mg/dL for *E. coli*, 203 mg/dL for *L. monocytogenes*, 152 mg/dL for *S. agalactiae*, 119 mg/dL for *H. influenzae*, 110 mg/dL for *C. neoformans*, and 62 mg/dL for *N. meningitidis* meningitis (Table 1).

Immunosuppression was more common in patients with cryptococcal meningitis, with 51.9% (14 out of 27) being immunosuppressed, compared to 12.2% (5 out of 41) of the patients with meningitis due to *S. pneumoniae*. In-patient mortality for cryptococcal meningitis was 33.3% (9 out of 27), whereas 19.5% (8 out of 41) for patients with meningitis due to *S. pneumoniae* (Table 1). We summarized the results as true positives (TPs), likely TP, or false positives (FPs) by the pathogen detected in Table 2. For the *C. neoformans* analyte, 88.9% (24 out of 27) of the results were classified as TPs, compared to 54.5% (6 out of 11) for *E. coli*. Likely TPs were more common in bacterial meningitis, 42% (37 out of 88) compared to 7.4% (2 out of 27) for *C. neoformans* (*P* < 0.001). Overall, 106 MEP results (92.2%) were classified as TP or likely TP. Among patients with likely TPs, 75.7% (28 out of 37) received oral or IV antibiotics before blood or CSF culture. In particular, 89.7% (seven out of eight) of the patients with *H. influenzae* had negative blood and CSF cultures, likely from prior antibiotic use.

**TABLE 2:** Clinical correlation of positive BioFire FilmArray meningitis/encephalitis panel (MEP) for bacterial and fungal organisms and CSF parameters

	*Streptococcus pneumoniae* *N* = 41 (%)	*Streptococcus agalactiae* *N* = 14 (%)	*Hemophilus influenzae* *N* = 16 (%)	*Escherichia* *coli* *N* = 11 (%)	*Listeria* *monocytogenes* *N* = 5 (%)	*Neisseria* *meningitidis* *N* = 1 (%)	*Cryptococcus neoformans* *N* = 27 (%)
True positives (TPs)	22 (53.7)	5 (35.7)	6 (37.5)	6 (54.5)	4 (80)	0	24 (88.9)
Likely true positives (likely TPs)	17 (41.4)	6 (42.9)	8 (50)	4 (36.3)	1 (20)	1 (100)	2 (7.4)
False positives (FPs)	2 (4.9)	3 (21.4)	2 (12.5)	1 (9)	0	0	1 (3.7)
Positive predictive value (PPV)	(95.1)	(78.6)	(87.5)	(90.9)	(100)	(100)	(96.3)
Sensitivity	100% (91–100%)	100% (71.5–100%)	100% (76.8–100%)	100% (62.9–100%)	100% (47.8–100%)	100% (2.5–100%)	100% (86.8–100%)
Specificity	100% (99.9–100%)	100% (99.9–100%)	100% (99.9–100%)	100% (99.9–100%)	100% (100–100%)	100% (100–100%)	100% (99.9–100%)
Positive predictive value	95.1% (83.5–99.4%)	78.6% (49.2–95.3%)	87.5% (61.7–98.4%)	90.9% (58.7–99.8%)	100% (47.8–100%)	100% (2.5–100%)	96.3% (81–99.9%)
Negative predictive value	100% (100–100%)	100% (100–100%)	100% (100–100%)	100% (100–100%)	100% (100–100%)	100% (100–100%)	100% (100–100%)

Although the case numbers are limited, patients with *L. monocytogenes* and *N. meningitidis* achieved 100% PPV. The PPV for the other bacterial analytes is arranged from higher to lower as follows: *S. pneumoniae* analyte (95.1%), *E. coli* (90.9%), *H. influenzae* (87.5%), and *S. agalactiae* (78.6%). Sensitivity, specificity, PPV, and NPV are shown in Table 3. Negative CSF bacterial and fungal cultures were observed in all negative Biofire MEP tests, and for samples positive for organisms not included in the panel indicating a specificity of 100% for these panel analytes.

**TABLE 3:** Sensitivity, specificity, PPV, and NPV of bacterial and cryptococcal analytes in the ME panel

	Cryptococcal analyte MEP (95% CI)	Bacterial analytes MEP (95% CI)
Sensitivity	100.0% (86.8–100.0%)	100.0% (95.5–100.0%)
Specificity	100.0% (99.9–100.0%)	99.9% (99.8–100%)
Positive predictive value	96.3% (81.0–99.9%)	90.9% (82.9–96.0%)
Negative predictive value	100.0% (100.0%)	100.0% (100.0%)

Nine out of 115 (7.8%) MEP results were classified as FPs compared to patients' clinical presentations, CSF cultures, and other test parameters. The analyte for *S. agalactiae* accounted for one-third (3 out of 9) of all the FP cases. Two FP results were attributed to *S. pneumoniae*, two to *H. influenzae*, and one case each to *E. coli* and *C. neoformans*. Clinical characteristics and outcomes of patients with FP Biofire MEP results with negative CSF Gram stain and cultures are shown in Table 4. The clinical symptoms and imaging studies for all FP patients were not consistent with meningitis. The data for CSF cell count and protein were not available for one patient. One patient with a positive MEP for *C. neoformans* had a nucleated cell count of 103 cells/µL, protein of 123 mg/dL, and glucose of 63 mg/dL. That patient was seen by the Pediatric Infectious Disease team who strongly suspected this was a FP result since the child had no signs or symptoms of meningitis. The patient had not left the hospital since birth and, therefore, would be unlikely to have encountered this pathogen. CSF cryptococcal antigen and India ink were also negative. Three of the nine FPs were treated with a full course of IV antibiotics. Two were not treated at all, and the rest were initially started on empiric antibiotics but were discontinued after extensive additional CSF analyses were negative.

**TABLE 4:** Clinical characteristics and outcome of patients with false positive Biofire ME panel with negative CSF Gram stain and cultures

No.	Organism	Age	Sex	Ethanicity	Presenting symptoms	Underlying illness	CSF WBC (0–5 cells/μL)	CSF protein (15-45 mg/dL)	CSF glucose (45–80 mg/dL)	Imaging findings	Received antibiotics prior to LP	Outcome	Interpretation of test result
*1*	*Streptococcus pneumoniae*	50d	M	W	Seizure-like activity	Femur fracture MRSA	3	80	47	No meningitis on MRI	No	Discharge	Treated for 10 days due to seizure
*2*	*Streptococcus pneumoniae*	17y	M	W	Optic neuritis	Chronic headache	2	38	61	No meningitis on MRI	No	Discharge	Treated despite the result being discounted as a probable FP
*3*	*Streptococcus agalactiae*	53y	F	W	Seizures	CKD, DM	8	44	157	No meningitis on MRI	No	Discharge	Treatment was stopped after one day of Empiric antibiotics
*4*	*Streptococcus agalactiae*	79y	M	Asian	Increased SOB, productive cough	Glioblastoma	1	33	61	Residual tumor in right cerebral hemisphere on CT head	Yes	Discharge	Treated for *Pneumocystis Jirovecii* pneumonia. Not treated for meningitis.
*5*	*Streptococcus agalactiae*	24y	M	W	Optic neuritis	Depression	1	23	60	No brain imaging	No	Discharge	Treatment was stopped after 1 day of Empiric antibiotics
*6*	*Hemophilus influenzae*	39y	F	W	Vague visual symptoms	Fungal endophthalmitis	2	35	76	No intracranial abnormalities on MRI	No	Discharge	Treatment was stopped after 2 days of Empiric antibiotics
*7*	*Hemophilus influenzae*	49y	F	W	Weakness, fatigue	Cholangiocarcinoma, left great toe osteomyelitis	1	15	62	T2 hyperintense supratentorial white matter lesions, consistent with multiple sclerosis	Yes	Discharge	Treatment for osteomyelitis. It was stopped after CSF culture was negative at 5 days.
*8*	*Escherichia coli*	49d	M	W and AA	Hypothermia	Preterm, RDS of prematurity	n/a	n/a	n/a	Unremarkable MRI of brain	Yes	Discharge	Failed bedside LP. Received 1 day of antibiotics prior to a successful LP by IR. Treated despite the result being discounted as a probable FP.
*9*	*Cryptococcal neoformans*	8d	M	W	Fever	s/p gastroschisis repair on Day 3	103	123	63	No head imaging	No antifungal	discharge	Discounted as FP, patient not treated

The comparison of CSF parameters of TPs and FPs is shown in Table 5. Higher CSF cell counts, high CSF protein, and low CSF glucose were seen in TP cases compared to FP cases. If MEP were not used in all ME cases in our center, 27.4% (29 out of 106) cases (excluding the FP) would not have had a pathogen definitively identified.

**TABLE 5:** Comparison of CSF WBC, glucose, protein levels between true positives (TP and likely TP) and false positives cases

Bacterial meningitis	True positives	False positives	*P* value
CSF cell count	(*n* =78)	(*n* = 6)	<0.001
Normal (0–5/µL)	2 (2.6%)	5 (83.3%)	
High (>5/µL)	76 (97.4%)	1 (16.6%)	
CSF protein	(*n* = 78)	(*n* = 7)	<0.001
Low (<15 mg/dL)	0 (0.0%)	0 (0.0%)	
Normal (15–45 mg/dL)	2 (2.6%)	6 (85.7%)	
High (>45 mg/dL)	76 (97.4%)	1/7 (14.3%)	
CSF glucose	(*n* = 77)	(*n* = 7)	0.003
Low (<45 mg/dL)	49 (63.6%)	0 (0.0%)	
Normal (45–80 mg/dL)	21 (27.3%)	6 (85.7%)	
High (>80 mg/dL)	7 (9.1%)	1 (14.3%)	

Cryptococcal meningitis	True positives	False positives	*P* value
CSF cell count	(*n* = 26)	(*n* = 1)	1.000
Normal (0–5/µL)	4 (15.4%)	0 (0.0%)	
High (>5/µL)	22 (84.6%)	1 (100.0%)	
CSF protein	(*n* = 26)	(*n* = 1)	1.000
Low (<15 mg/dL)	0 (0.0%)	0 (0.0%)	
Normal (15–45 mg/dL)	2 (7.7%)	0 (0.0%)	
High (>45 mg/dL)	24 (92.3%)	1 (100.0%)	
CSF glucose	(*n* = 26)	(*n* = 1)	0.407
Low (<45 mg/dL)	16 (61.5%)	0 (0.0%)	
Normal (45–80 mg/dL)	10 (38.5%)	1 (100.0%)	
High (>80 mg/dL)	0 (0.0%)	0 (0.0%)	

## DISCUSSION

Several comprehensive studies have rigorously evaluated the MEP performance in diagnosing CNS infections. These investigations consistently underscore the panel’s high sensitivity in identifying bacterial and viral pathogens, boasting rapid results crucial for prompt clinical decision-making. Moreover, the MEP significantly enhances pathogen identification in meningitis cases, particularly when traditional methods falter due to negative Gram stains (7, 13, 14). One meta-analysis with eight studies including 3,059 patients reported a sensitivity and specificity of 90% (95% CI: 86–93%) and 97% (95% CI: 94–99%), respectively. The PPV of the MEP was 85.1%, and the NPV was 98.7%. The highest proportion of FPs was observed for *S. pneumoniae* (17.5%), followed by *S. agalactiae* (15.4%). *C. neoformans/gattii* had the highest proportion of false negative determinations in this study (11). Another meta-analysis with 15 studies on bacterial meningitis found a sensitivity of 92.1% and specificity of 99.2%. The FP rate was 9.4% based on laboratory and clinical analysis to complement the MEP PCR results. It resulted in some cases adjudicated as a CNS infection. The authors concluded that the true FP rate for bacteria might be between 9.4% and 46.4% (15). In a case series, Waldrop et al. described three FP cases for *S. agalactiae*, two for *H. influenzae*, and one for *E. coli*. (16) In one study, 7 out of 27 (26%) positive bacterial MEP results were deemed unlikely to be clinically significant because CSF profiles and clinical presentations were inconsistent with bacterial meningitis (16).

In our study, the PPV of the molecular testing for bacterial and fungal pathogens varied based on the identified organism, ranging from 78.6% to 100%. One-third of the FP results were *S. agalactiae*, and FP results were more common in the older populations. Of the 115 patients tested, 9 (9.1%) with positive results were deemed clinically improbable since the patients' clinical manifestations and CSF parameters did not suggest meningitis. Our study observed the highest rate of FPs for *S. agalactiae* (21.4%), followed by *H. influenzae* (12.5%). No false negative *C. neoformans* results were seen in our case series. Detection of a pathogen, although suggestive, is not diagnostic of infection. CSF parameters and clinical manifestation should be considered to determine the true etiology.

Epidemiology of CNS infections varies by patient population. In a study in England, the leading community-acquired pathogen causing meningitis was *S. pneumoniae* (811 out of 4,073, 19.9%), and its incidence increased significantly from 2012 to 2019. In infants, *S. agalactiae* was prominent in <3 months, followed by *N. meningitidis* and *S. pneumoniae* in 3- to 11-month-olds (17). Our study also showed *S. pneumoniae* was the main pathogen for bacterial meningitis. However, *E. coli* and *S. agalactiae* were the two common causes of bacterial meningitis in neonates in our study. Early neonatal disease (within 1 month of birth) was not very common in our patient population and occurred only in 36.4% (4 out of 11) cases of *E. coli* meningitis and 21.4% (3 out of 14) of *S. agalactiae* meningitis. The median age was younger in patients with *E. coli* (5 weeks) and *S. agalactiae* (9 weeks) but older in patients with *S. pneumoniae* (47 years) and *C. neoformans* (49 years). Patients with *S. pneumoniae* meningitis had the highest median CSF cell count, protein, and lowest CSF glucose. It had higher in-patient mortality among patients with bacterial meningitis. The mortality of patients with *S. pneumoniae* meningitis was not statistically significant compared to those with cryptococcal meningitis.

Interestingly, 27.4% (29 out of 106) of our cases (excluding the FP) would not have had a pathogen definitively identified if MEP was not used. Among patients with likely TPs results, 75.7% (28 out of 37) received oral or IV antibiotics at least 24 h before blood or CSF cultures. Empiric antibiotics were stopped after CSF analysis were reported in four out of nine FPs (44.4%). Positive test results allowed for drug optimization and duration of therapy. Fast turnaround times of MEP (approximately 1 h) could potentially decrease the number of other diagnostic tests, length of stay, and length of empiric antimicrobial therapy (18). The CSF culture yield is low at 30–50%, and it usually takes 2–5 days for a bacterial culture to be completed. Furthermore, culture can be falsely negative if the organism is fastidious or the patient has recently been exposed to antibiotics. Research has indicated that similar to decreasing culture positivity, pretreatment with antibiotics may decrease the rate of bacterial detection in CSF by PCR (19). Other investigators analyzing the clinical impact of the MEP on antimicrobial use and duration of therapy found that prior antibiotic use can have implications for the performance of the MEP (6). In one study, 7 out of 12 CSF samples were positive only by ME PCR panel obtained from infants who had received prior antibiotic treatment (20). In our study, seven of nine FPs (77.8%) received antibiotics.

*C. neoformans and C. gattii* can be falsely negative by MEP (9). MEP detected 84.2% (32 out of 38) cryptococcal antigen-positive (CrAg) specimens. In one study, the sensitivity and specificity were 83.8% (95% CI: 68.0–93.8%) and 99.9% (95% CI: 99.6–100.0%) compared to CSF CrAg testing (9). However, all five CrAg-positive, MEP, and culture-negative specimens were obtained from previously treated CM patients. Our study compared MEP with the CSF and blood culture, not with CrAg. There was a high rate of blood culture positivity (88.9%) for patients with cryptococcal meningitis compared to bacterial pathogens, where the blood culture positivity rates ranged from 33.3% to 50% depending on the organism. In contrast to bacterial cultures, fungal growth is not altered by the use of antibacterial agents. Antifungals are not usually administered until a definitive diagnosis of cryptococcal meningitis is made, so these cultures are not usually inhibited, leading to a higher culture positivity rate for these patients.

While this study is limited by its being a retrospective study in a single institution, its strength lies in its extensive scope and duration, and the number of patents included contributing to its comprehensive analysis. The time frame spans the entire period of use of this panel in our facility, nearly 6 years, and includes all 6,943 patients tested. Including children and adults allows for the detection of significant pathogens across the human lifespan in the patient population in Central Kentucky.

In conclusion, the BioFire FilmArray ME molecular panel demonstrated high sensitivity and specificity compared to CSF and blood cultures and other clinical and testing parameters. A small percentage of positive MEP results were clinically improbable and were classified as FPs. The correlation of MEP results with multiple CSF parameters and clinical presentation is essential for the arrival of an accurate diagnosis. A rapid MEP test may be particularly valuable in cases where patients have received prior antibiotic treatment.
